# PBRM1 deficiency enhances PD1 immunotherapeutic sensitivity via chromosomal accessibility in colorectal cancer

**DOI:** 10.7150/thno.100793

**Published:** 2025-02-18

**Authors:** Rui Li, Jie He, Chaoqun Liu, Zesheng Jiang, Jiasheng Qin, Kun Liang, Zhuocheng Ji, Liang Zhao

**Affiliations:** 1 Department of Pathology, Shunde Hospital of Southern Medical University, Foshan, China.; 2 Department of Pathology & Guangdong Province Key Laboratory of Molecular Tumor Pathology, Basic Medical College, Southern Medical University, Guangzhou, China.; 3 Department of Pathology, Nanfang Hospital, Southern Medical University, Guangzhou, China.; 4 General Surgery Center, Department of Hepatobiliary Surgery II, Guangdong Provincial Research Center for Artificial Organ and Tissue Engineering, Guangzhou Clinical Research and Transformation Center for Artificial Liver, Institute of Regenerative Medicine, Zhujiang Hospital, Southern Medical University, Guangzhou, China.

**Keywords:** Colorectal cancer, PBRM1, Chromosome accessibility, Exosome, PD1 immunotherapeutic

## Abstract

**Rationale:** Tumor cell epigenetics, especially chromosome accessibility, has been reported to be closely related to the tumor immune landscape and immunotherapy. However, the exact mechanism remains unknown.

**Methods:** Whole-exome sequencing was used to analyze 13 colorectal tumor samples treated with PD1 immunotherapy. The assays for transposase-accessible chromatin using sequencing (ATAC-seq) and RNA sequencing were used to detect tumor cells' chromosome accessibility status and screen regulatory pathways.

**Results:** Polybromo-1 (PBRM1) was one of the 12 genes with the highest frequency of somatic mutations associated with immunotherapy sensitivity. PBRM1/Pbrm1 deficiency in colorectal cancer promoted PD-1 immunotherapy sensitivity and chemotaxis of CD8^+^ T and NK cells in the microenvironment *in vivo* and *in vitro.* ATAC sequencing revealed that deletion of Pbrm1, a critical component of the SWI/SNF complex, increased chromosomal accessibility in tumor cells and triggered the release of cytokines, such as CCL5 and CXCL10, by activating the NF-κB signaling pathway. Application of ACBL1, a PROC inhibitor of PBRM1, in BALB/C mice or colorectal patient-derived tumor organoids (PDTOs) significantly promoted the sensitivity to PD1 antibody immunotherapy.

**Conclusions:** Our study established that PBRM1/Pbrm1 deficiency was positively correlated with PD1 immunotherapeutic sensitivity in colorectal cancer. The underlying molecular mechanisms involved regulation of chromosome accessibility, activation of the NF-κB signaling pathway, and immune cell infiltration in the microenvironment. These findings identify potential molecular targets for enhancing immunotherapy for colorectal cancer.

## Introduction

Colorectal cancer (CRC) is a malignant tumor that poses a significant threat to human survival and health worldwide 1. With the advances in immunology in recent years, transforming a "cold tumor" into a "hot tumor" with higher immunogenicity and better immunotherapy effect is an active area of research to improve the benefits of clinical immunotherapy 2.

It has been reported that aberrant regulation of the epigenome drives anomalous transcriptional programs that contribute to cancer development and progression and are also involved in tumor immunogenicity and the immune microenvironment 3. Chromosome accessibility plays an important role in tumorigenesis, progression, and treatment. A survey of 410 tumor samples spanning 23 cancer types identified genetic risk loci for cancer susceptibility as active DNA regulatory elements that interact with each other to accomplish immune evasion of cancer 4.

The switching sucrose non-fermenting (SWI/SNF) complex regulates chromatin accessibility for transcription factors. It has an average mutation rate of 25%, which is lower than the 26% mutation rate of P53 and the second highest among all genes 5. Polybromo 1 (PBRM1), a tumor suppressor gene that encodes the BAF180 protein, is a specific subunit of the PBAF complex. PBRM1 is one of the most frequently altered genes in cancer 6. Deleterious PBRM1 mutations have been found in 28%-55% of clear cell renal cell carcinomas (ccRCC) 7. Several other aggressive malignancies also harbor PBRM1 defects, including 11-59% chordomas, 12-23% cholangiocarcinomas, 7-20% mesotheliomas, 12% endometrial carcinomas, 3% non-small cell lung cancers (NSCLC), and 5% colorectal cancer (CRC)^8^. More importantly, recent studies have shown that mutations in PBRM1 are closely associated with poor tumor prognosis and immunotherapeutic sensitivity.

The pan-cancer landscape of CD274 (PD-L1) rearrangements in 283,050 patient samples demonstrated a positive correlation between mutations in PBRM1 and PD-L1 expression and tumor mutational burden (TMB) 9. In tumors such as NSCLC 10 and ccRCC 11, individuals with somatic PBRM1 mutations had upregulated treatment sensitivity to PD1/PDL1 inhibitors. PBRM1 is only present in PBAF morphology, and its absence could promote ccRCC and correlate with immunotherapeutic susceptibility to multiple tumors. In colorectal cancer, the low expression of PBRM1 was an independent risk factor for poor patient prognosis 12. In another cohort study that included 5,143 patients with tumors, whole-exome sequencing showed mutations of PBRM1 in 264 patients, 52% of whom had gastrointestinal tumors 13. This study also demonstrated T-cell effector genes (CD8B, CD40LG), central memory marker genes (CD27, CCR7), and mature B-cell marker genes (CD20, CD38, CD79, IRF4) were up-regulated in cases with PBRM1 mutations compared with the wild group.

Although PBRM1 showed a high frequency of mutations in colorectal cancer and is strongly associated with poor prognosis, the role and potential mechanisms of the SWI/SNF complex and PBRM1 mutations in immunotherapy in colorectal cancer remain unclear. In this study, we screened PBRM1, which is associated with PD1 therapeutic sensitivity in CRC, by whole-exome sequencing and found that its deficiency can regulate chromosome accessibility and induce infiltration of CD8^+^ T cells and Natural Killer (NK) cells in the microenvironment. Our findings provide groundwork for enhancing the immunotherapeutic sensitivity in CRC.

## Materials and Methods

### Cell culture

The normal human colonic epithelial cell line NCM460, colorectal cancer cell lines SW480, SW620, HCT116, LS174T, and HT29, and mouse colorectal cancer cell line CT26 were obtained from the Cell Bank of the Chinese Academy of Sciences (Shanghai, China) and maintained as previously described 14. All cells were cultured in RPMI-1640 (Hyclone, Logan, UT, USA) supplemented with 10% fetal bovine serum (Gibco-BRL, Invitrogen, Paisley, UK) at 37° C and 5% CO_2_ humidity.

### CRC patient-derived tumor organoids culture

CRC patient-derived tumor organoids (PDTOs) were constructed as previously described 15. Patients were not involved in the design, conduct, reporting, or dissemination plans of our research. Fresh patient tumor tissues were mechanically minced, digested using Gentle Cell Dissociation Reagent (STEAMCELL, 07174) at 37° C for 1 h, and filtered through a 70 µm mesh. The organoids were mixed with Matrigel (356231; Corning), and the culture medium (bioGenous, K2103-CR) was added after the Matrigel solidified and refreshed every 2 days.

Gene editing of the PDTOs was performed according to established protocols in the literature. The viral supernatant was concentrated by high-speed centrifugation at 80,000 × g for 2 h at 4^o^ C. Approximately 48 h after viral infection, organoids were selected in a culture medium containing 2 µg/mL puromycin for 7 consecutive days. PDTOs were seeded in 96-well plates and incubated. PDTO growth was evaluated using the CellTiter-Glo® 3D Cell Viability Assay (Promega, G9683).

### Animals

All animal experiments were approved by the Institutional Animal Care and Use Committee of Southern Medical University (Guangzhou, China). Female BALB/c mice and female BALB/c nude mice (3-5 weeks old, 14-16 g) obtained from Nanfang Medical University Experimental Animal Center were housed in SPF animal rooms with free access to sterilized food and water. BALB/c mice were divided into four groups: (1) control group injected with sgNC-CT26 cells, (2) knockout group injected with sgPBRM1-CT26 cells, (3) control group injected with sgNC-CT26 cells and orally administered pharmacologic grade Anti-PD1, and (4) knockout group injected with sgPBRM1-CT26 cells and orally administered pharmacologic grade Anti-PD1 (BE0146; BioXcell, 200 µg/ip, dissolved in 5% saline) from 1 week after the injection to the end of sample harvest. Female BALB/c-hPD1 mice were obtained from GemPharmatech Co., divided into four groups, and injected with Opdivo (200 µg/ip, dissolved in 5 % saline).

### Statistical analysis

Data were analyzed using the SPSS statistic software version 19.0 (SPSS; Chicago, IL, USA). RT-PCR was performed using Student's *t*-test and one-way ANOVA. Kaplan-Meier plots were used to estimate the prognostic relevance of PBRM1 in univariate analysis. The Cox proportional hazards test was used for multivariate analysis. Statistical significance was defined as P < 0.05.

## Results

### PBRM1 deficiency was positively associated with CRC immunotherapy sensitivity

**Figure 1:**
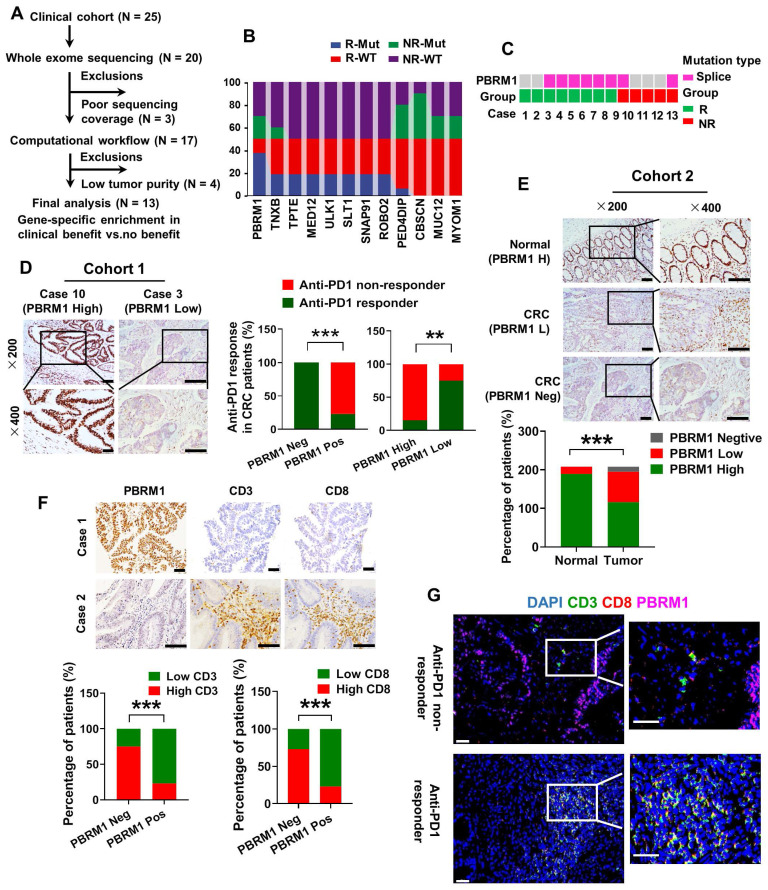
** PBRM1 deficiency is positively associated with CRC immunotherapy sensitivity.** (A) Selection of the validation cohort. (B) Interactive ribbon bar graph showing the 12 genes with the highest difference in mutation frequency in the immunotherapy-sensitive versus non-sensitive groups of human colorectal cancer patients. (C) Truncating alterations in PBRM1 and response to anti-PD-1 therapies. Colored boxes indicate samples with truncating mutations in PBRM1, while gray denotes samples without PBRM1 truncating mutations. (D) Left panel: Immunohistochemistry confirmed that missense LOF denotes a missense mutation detected by targeted sequencing in 25 clinical colorectal cancer samples treated with PD-1 immunotherapy. Right panel: Statistical graph showing the relationship between PBRM1 LOF and PBRM1 protein expression and immunotherapy sensitivity. The double asterisk (**) indicates P < 0.01. The quadra asterisk (****) indicates P < 0.0001. Scale bar represents 50 µm. (E) Immunohistochemical analysis of PBRM1 expression in 208 colorectal cancer tissues with paired normal tissue. Scale bar represents 50 µm. (F) Immunohistochemical analysis of the association between PBRM1 expression and the abundance of lymphocyte infiltration in the mesenchyme in 208 colorectal cancer tissues with paired normal tissues. Scale bar represents 50 µm. (G) Multiplex immunofluorescence analysis of PBRM1 expression in relation to lymphocyte infiltration in tumor mesenchyme in 25 clinical colorectal cancer samples treated with PD-1 immunotherapy. Scale bar represents 30 µm.

To identify genomic alterations in CRC that correlate with the response to anti-PD-1 monotherapy, we performed whole exome sequencing (WES) of metastatic CRC samples from 13 patients with complete clinical prognostic information (Figure 1A). As shown in Figure 1B, we screened 12 genes with the highest frequency of loss-of-function (LOF) alterations, including truncating alterations, deep deletions, and other missense mutations, comparing the PD1 immunotherapy-sensitive and -insensitive groups. PBRM1, which encodes a subunit of the SWI/SNF chromatin remodeling complex (the PBAF subtype), sustained a high frequency of mutations in the PD1 immunotherapy-sensitive group (75%, 6/8) compared with the insensitive group (40%, 2/5) (Figure 1C). We used immunohistochemical staining to examine 25 colorectal cancer samples treated with PD-1 immunotherapy and found a strong association between the lack of protein expression and LOF of PBRM1 with immunotherapy sensitivity (Figure 1D). All four samples with complete LOF of PBRM1 showed strong sensitivity to PD1 immunosuppressive therapy and were independent of microsatellite status (Figure S1). Besides, we analyzed data on high-frequency mutations in samples with PBRM1 mutations and found no specific concomitant mutant genes (Figure S2).

Next, we used the TIMER database to analyze the cancer data from The Cancer Genome Atlas (TCGA) for PBRM1 expression levels. As shown in Figure S3, there was a significant decrease in PBRM1 RNA expression in cancer tissues compared to normal tissues, including bladder urothelial carcinoma (BLCA), breast invasive carcinoma (BRCA), colon adenocarcinoma (COAD), and lung adenocarcinoma (LUAD). The immunohistochemical analysis included 208 paired colorectal cancer samples with complete clinical data. The results are shown in Figure 1E. Compared with normal tissues, PBRM1 was expressed at a low level in colorectal cancers and showed a complete deletion mutation in 28.4% (59/208) of colorectal cancers. Kaplan-Meier survival analysis of a previously published CRC dataset (TCGA Pan-cancer atlas, n = 594) revealed that PBRM1 deficiency was closely correlated with patients' survival rate and life expectancy (Figure S4). Also, immunohistochemical staining of 61 human colorectal cancer tissues similarly showed that PBRM1 expression was not associated with the expression of P53, BRAF-V600E, and KRAS in CRC tissues, commonly mutated genes in colorectal cancer (Figure S5).

TIMER database analysis also showed that the infiltration abundance of immune cells, such as CD8^+^ T cells, neutrophils, and dendritic cells, in the tumor mesenchyme was negatively correlated with PBRM1 expression (Figure S6). Immunohistochemical staining of 208 human colorectal cancer tissues similarly showed that PBRM1 expression inversely correlated with the abundance of CD8^+^ T cell infiltration (Figure 1F). Furthermore, fluorescence staining of 25 colorectal cancer samples treated with PD-1 immunotherapy exhibited a significant abundance of infiltrating CD8^+^ T cells in PBRM1-deficient tissues compared to the PBRM1 wild-type group. This observation suggested that PBRM1 deficiency is closely associated with anti-PD1 immunotherapeutic sensitivity in colorectal cancer and may correlate with T-cell infiltration in the tumor mesenchyme (Figure 1G).

### PBRM1 deficiency promoted colorectal cancer sensitivity to anti-PD1 therapy* in vivo*

**Figure 2:**
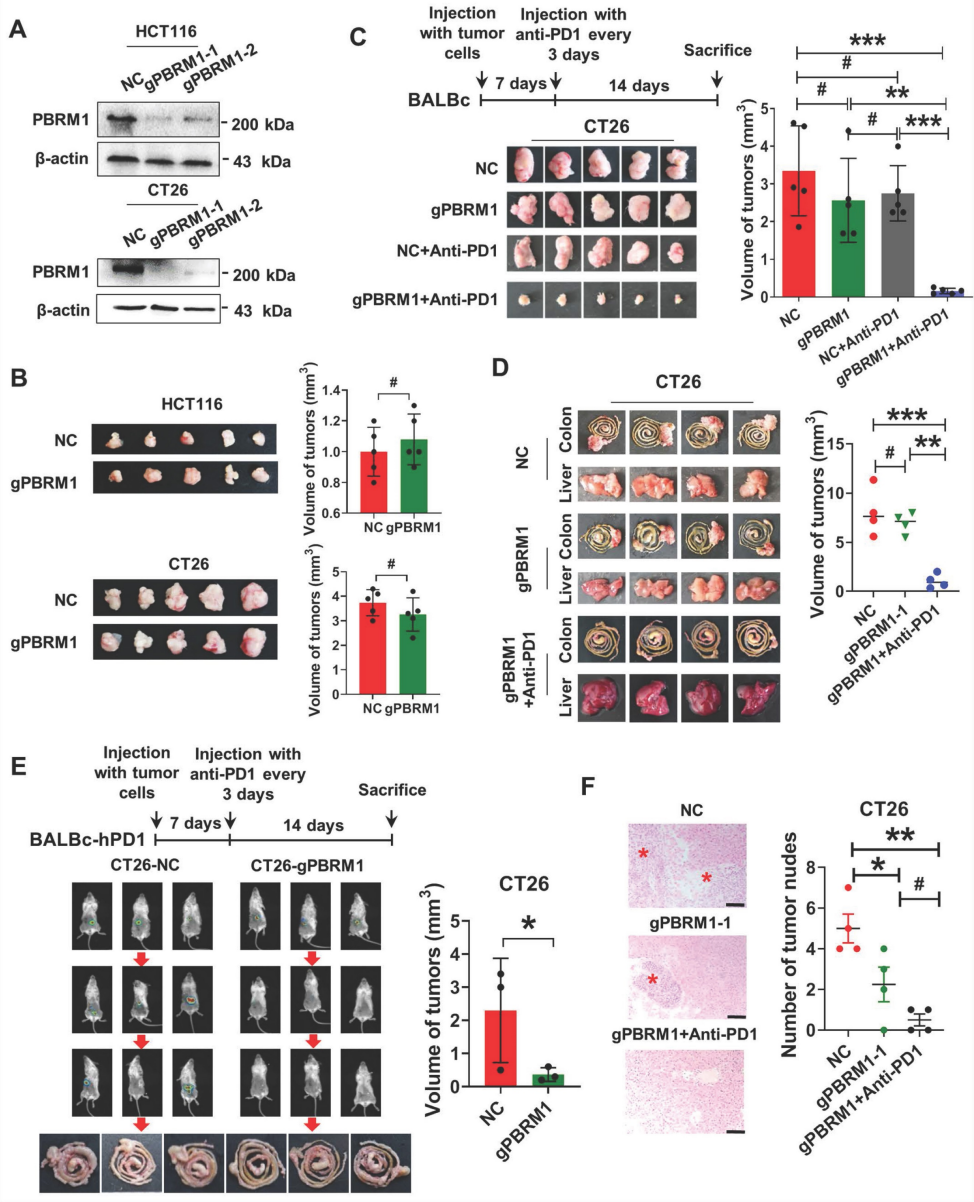
** PBRM1 deficiency promotes colorectal cancer sensitivity to anti-PD1 therapy *in vivo*.** (A) Western blot analysis showed that PBRM1 knockout cells were constructed. in HCT116 cells (upper panel) and CT26(lower panel) cells. (B) sgNC and sgPBRM1 HCT116 cells (upper panel) and CT26(lower panel) cells were injected subcutaneously into the backs of nude mice to access tumor growth. The representative figure of the tumors was shown. The right bar represents the weight of the tumors, and the asterisk (#) indicates P > 0.05. (C) sgNC and sgPBRM1 CT26 cells were injected subcutaneously into the backs of BALB/C mice with or without Anti-PD1 (200 µg/ip) to evaluate tumor growth. A representative figure of the tumors was shown. The histogram was on the right, and the (#) indicates P > 0.05. The double asterisk (**) indicates P < 0.01. The quadra asterisk (***) indicates P < 0.001. (D) sgNC and sgPBRM1 CT26 cells were injected into the cecum mesentery of BALB/C mice with or without Anti-PD1 (200 µg/ip) to assess the weight of *in situ* tumor and the number of liver metastases. The histogram was on the right. (E) *In vivo*, imaging results showed the size of the cecum *in situ* tumors one week after sgNC, and sgPBRM1 CT26 cells were injected into the BALBc-hPD1mouse cecum mesentery after administration of the Opdivo (200 µg/ip). The histogram was on the right, and the asterisk (*) indicates P < 0.05. (F) HE staining showed the number of liver metastases corresponding to panel D. Scale bar represents 50 µm.

We investigated the effect of PBRM1 mutations on the biological function of tumor cells using the CRISPR-Cas9 gene editing system to introduce deletion mutations in human and mouse colorectal cancer cells, HCT116 and CT26. The successful construction of the mutant strains HCT116- gPBRM1-1 and CT26- gPBRM1-1 was evident by Western blotting (Figure 2A). However, *in vitro* CCK8 or plate cloning (Figure S7) and *in vivo* subcutaneous tumor assays in BALB/C mice (Figure 2B) showed that PBRM1 mutations did not affect the proliferation of colorectal tumor cells.

We clarified the effect of PBRM1 on the sensitivity of colorectal tumors to anti-PD1 immunotherapy by constructing a subcutaneous tumor model in BALB/c mice. After 14 days of PD-1 antibody treatment (200 µg/ip), the subcutaneous CT26-gPBRM1-1 tumor volume in mice was not significantly changed compared with the control group (Figure 2C). However, the tumor volume and the number of hepatic metastatic foci in the PBRM1 mutant group were significantly reduced after administering the PD-1 antibody (Figure 2D and Figure 2F). To further examine the effect of the PBRM1 mutation on the therapeutic sensitivity of PD1-1 antibody in colorectal tumor cells, we introduced humanized PD-1 mice (BALBc-hPD1). As displayed in Figure 2E, the PBRM1 mutant group treated with Opdivo (200 µg/ip) showed a significant reduction in the volume of their cecum primary tumors compared to the control group. This observation suggested that deletion mutations of PBRM1 in tumor cells do not affect the proliferative capacity of colorectal cancer cells *in vitro*; however, they can enhance the therapeutic sensitivity of colorectal cancer cells to PD-1 immunotherapy.

### PBRM1 deficiency promoted CD8^+^ T and NK cell activation *in vivo* and *in vitro*

**Figure 3:**
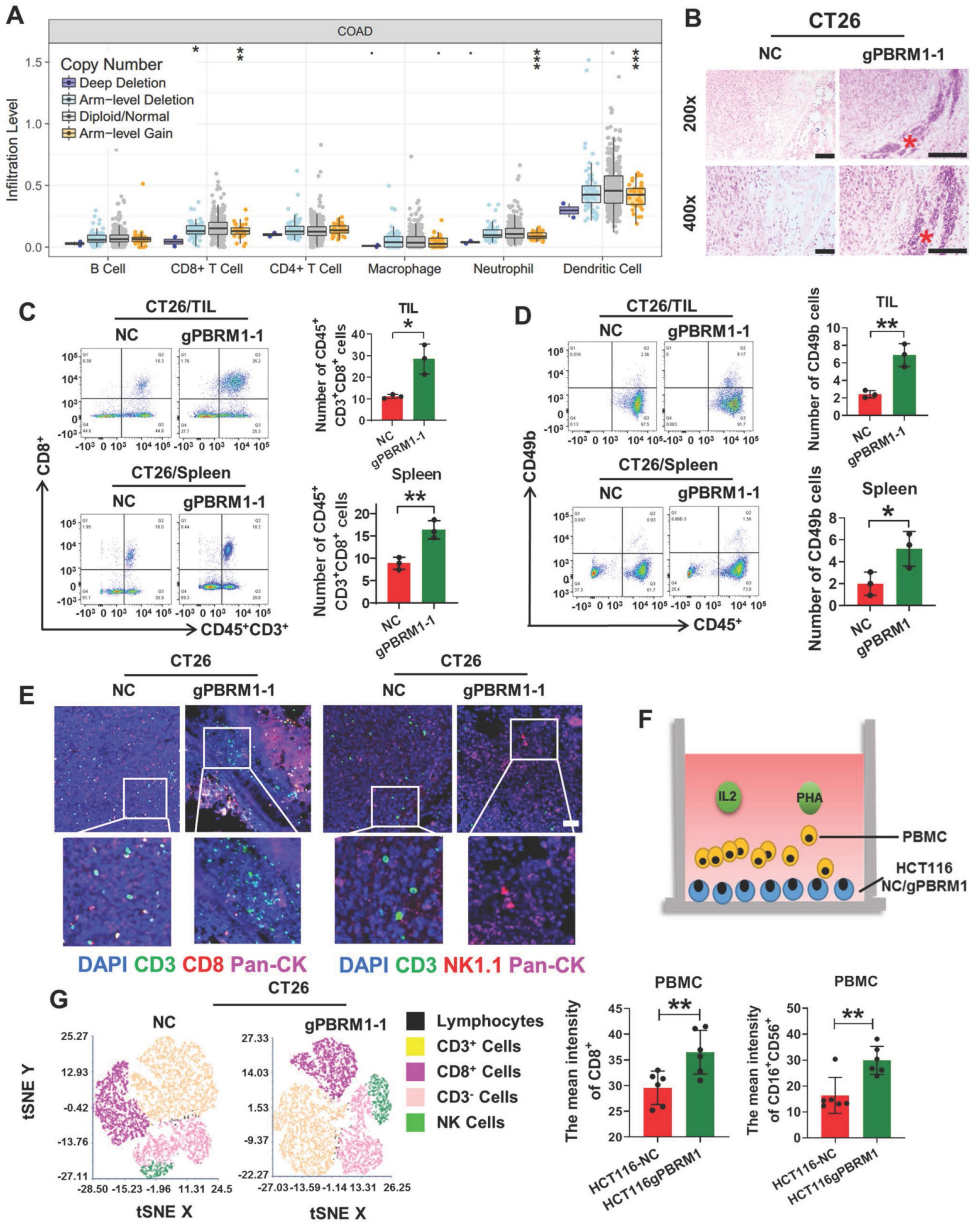
**PBRM1 mutation promotes CD8^+^ T and NK cell activation *in vivo* and *in vitro*.** (A) TIMER database analysis of the association between PBRM1 mutation type and tumor mesenchymal immune cell infiltration. (B) HE analysis of immune cells in BALB/C mouse tumor tissues injected with sgNC or sgPBRM1 CT26 cells. Scale bar represents 50 µm. (C) and (D): Proportion of CD8^+^T cells (C) and NK cells(D) of tumor-infiltrating lymphocytes and splenic lymphocytes in tissues of mice injected with sgNC or sgPBRM1 CT26 cells *in situ* via the cecum as revealed by flow cytometric assay. (E) Representative figures of immunofluorescence for tumor-infiltrating lymphocytes in tissues of mice injected with sgNC or sgPBRM1 CT26 cells *in situ* via the cecum. (F) The schematic diagram shows a co-culture system of peripheral blood mononuclear cells (PBMC) and CRC cells. (G) Left panel: T-distributed Stochastic Neighbor Embedding(t-SNE) indicating the proportion of immune cells in PBMC after co-culture with tumor cells, respectively. Right panel: Bar charts show the proportion of CD8-positive T cells and NK cells in PBMC after co-culture with HCT116-NC and HCT116-sgPBRM1. The double asterisk (**) indicates P < 0.01.

TIMER database analysis showed that PBRM1 expression was correlated with tumor mesenchymal lymphocyte infiltration. Also, the insertion or deletion of somatic mutations, especially point mutations in patients with colorectal cancer, was proportional to the number of infiltrating tumor-infiltrating lymphocytes, such as CD8^+^ T cells, neutrophils, and dendritic cells (Figure 3A). Therefore, we confirmed by HE staining that lymphocyte infiltration was significantly higher in the mesenchyme of *in situ* tumor tissues in the PBRM1-deficient group than in the control group (Figure 3B). Tumor-infiltrating lymphocytes of mice 14 days after seeding were used for flow cytometry. CD8+ T (Figure 3C) and natural and NK cells (Figure 3D) increased significantly compared with the control group. Similar results were obtained using multiple immunofluorescence assays (Figure 3E). As shown in Figure 3F schematic, flow cytometry was performed after co-culturing peripheral blood lymphocytes (PBMCs) with HCT116 mutant cell lines. After co-culturing with sgPBRM1-HCT116 cells for 72 h, the number of CD8^+^ T and NK cells in PBMCs was significantly increased compared to that in the NC group (Figure 3G), demonstrating that PBRM1 deficiency in CRC cells promotes chemotaxis of CD8^+^ T and NK cells in the mesenchyme.

### Pbrm1 mutation regulated chromosome accessibility and activated the NF-κB signaling pathway in CRC cells

**Figure 4:**
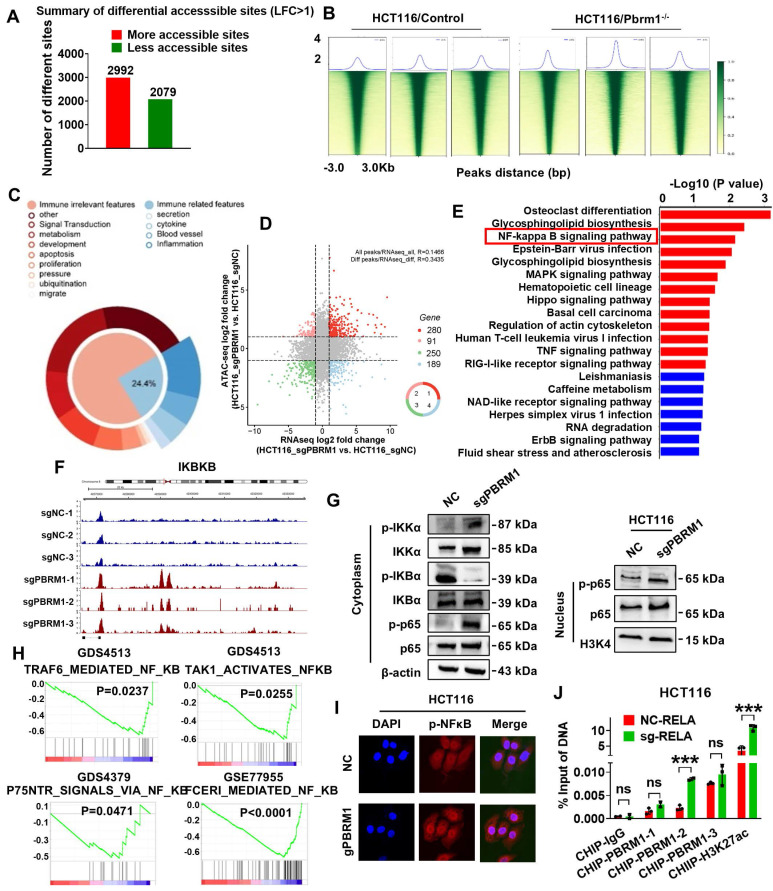
**Pbrm1 mutation regulates chromosome accessibility and activates the NF-κB signaling pathway in CRC cells.** (A). Genome-wide analysis of differentially accessible chromatin sites (|log2 fold change| >0.5) in control versus Pbrm1-deficient HCT116 tumor cells. (B) Chromatin accessibility heat maps for all sites. Aggregated reads within 2kb of the center of differentially accessible regions are shown above heat maps. (C) The circular sector plot shows ATAC-seq combined with RNA-seq analysis of top1000 differential genes from sgPBRM1 versus sgNC HCT116 cells. (D) The four-quadrant graph shows differential gene expression for ATAC-seq and RNA-seq combined results from sgPBRM1 versus sgNC HCT116 cells. Dots in red represent 280 upregulated genes (log2(FC) > 1 and adjusted p-value < 0.05), and dots in blue represent 189 downregulated genes (log2(FC) < -1 and adjusted p-value < 0.05) in sgPBRM1 versus sgNC HCT116 cells. (E) A dot map showing the top 20 terms in the Kyoto Encyclopedia of Genes and Genomes (KEGG) analysis of differential genes in sgPBRM1 versus sgNC HCT116 cells. n = 3 biologically independent samples per group. Statistical analysis was performed using modified Fisher's exact tests. (F) IGV plots depicting ATAC-Seq signals at selected gene loci. Peak height is positively correlated to accessibility. Genome-scale is included, and the y-axis is consistent for all loci (G) Western blot analysis of NFκb pathway members in nuclear and cytoplasm of sgPBRM1 and sgNC HCT116 cells. (H) Gene-set enrichment analysis (GSEA) plots for NFκB-regulated genes using Pbrm1 gene signature in human tumor database. p values are calculated using Kolmogorov-Smirnov tests. NES: normalized enrichment score. (I) IF assay was used to visualize the nucleus translocation of p-NFκb in sgPBRM1 and sgNC HCT116 cells. (J) The results of CHIP-qPCR showed the binding site on the RELA promoter with either the PBRM1 antibody, positive control (H3K27ac), or negative control (IgG) in HCT116 cells.

As an important component of the SWI/SNF complex, PBRM1 is thought to be closely associated with chromosomal accessibility. Therefore, we performed ATAC-sequence to directly assess chromatin accessibility in Pbrm1 deficient and control HCT116 tumor cells. As presented in Figure 4A, a substantially larger number of genomic sites were accessible in Pbrm1-deficient than in control HCT116 cells, and the peak of Pbrm1^-/-^ cells was significantly increased (Figure 4B). We then analyzed the top 1000 entries in the loci where chromosome openings occurred and found that 24.4 % were associated with natural immunity (Figure 4C). Combined with the RNA sequencing results, 280 genes showed chromosomal accessibility and increased transcription at the RNA level (Figure 4D). RNA sequencing results also showed that deletion of PBRM1 did not affect the transcript levels of other genes in the PBAF complex, including BRD7, ARID2, SMARCA2, and SMARCA4 (Figure S8).

Kyoto Encyclopedia of Genes and Genomes (KEGG) was used to predict the functional role of the differentially expressed genes (DEGs). Pbrm1 mutation was associated with cellular pathways, such as tumor cell vesicle regulation, sphingomyelin metabolism, NF-κB, MAPK, and P53. Motif and target gene prediction analysis suggested that these sites were highly enriched in IKBKB and RELA motifs (Figure 4F). Thus, the inactivation of Pbrm1 enhanced chromatin accessibility for transcription factors at promoters and enhancers of the NF-κB pathway, an evolutionarily conserved regulator of immune and inflammatory responses.

Western blot analysis indicated a stimulatory role for the PBRM1 mutation in the nuclear translocation of p65 and cytoplasmic translocation of IκB kinase alpha (IKK-α) (Figure 4G). Immunofluorescence analysis also showed that the PBRM1 mutation in HCT116 cells promoted the nuclear import of p-NFκB (Figure 4I). We also performed gene set enrichment analysis to identify the known gene sets/pathways. Analysis of the GSEA database revealed that PBRM1 was negatively correlated with the NF-κB signaling pathway (Figure 4H). To further validate whether PBRM1 binds to RELA to regulate the NF-κB signaling pathway, we performed promoter scanning in the JASPAR database and identified three probable PBRM1 binding sites within the RELA promoter region (Predicted sequence: GGGAGTTTCC, GGGTCTTTCC, AGGACTTTCC). These sites overlapped with the binding sites identified through ChIP-qPCR analysis, validating the direct binding of PBRM1 to the GGGTCTTTCC sequence in the RELA promoter in HCT116 cells (Figure 4J).

### PBRM1 deficiency-induced cytokine secretion promoted CRC cells' sensitivity to anti-PD1 therapy via the NF-κB signaling pathway

**Figure 5:**
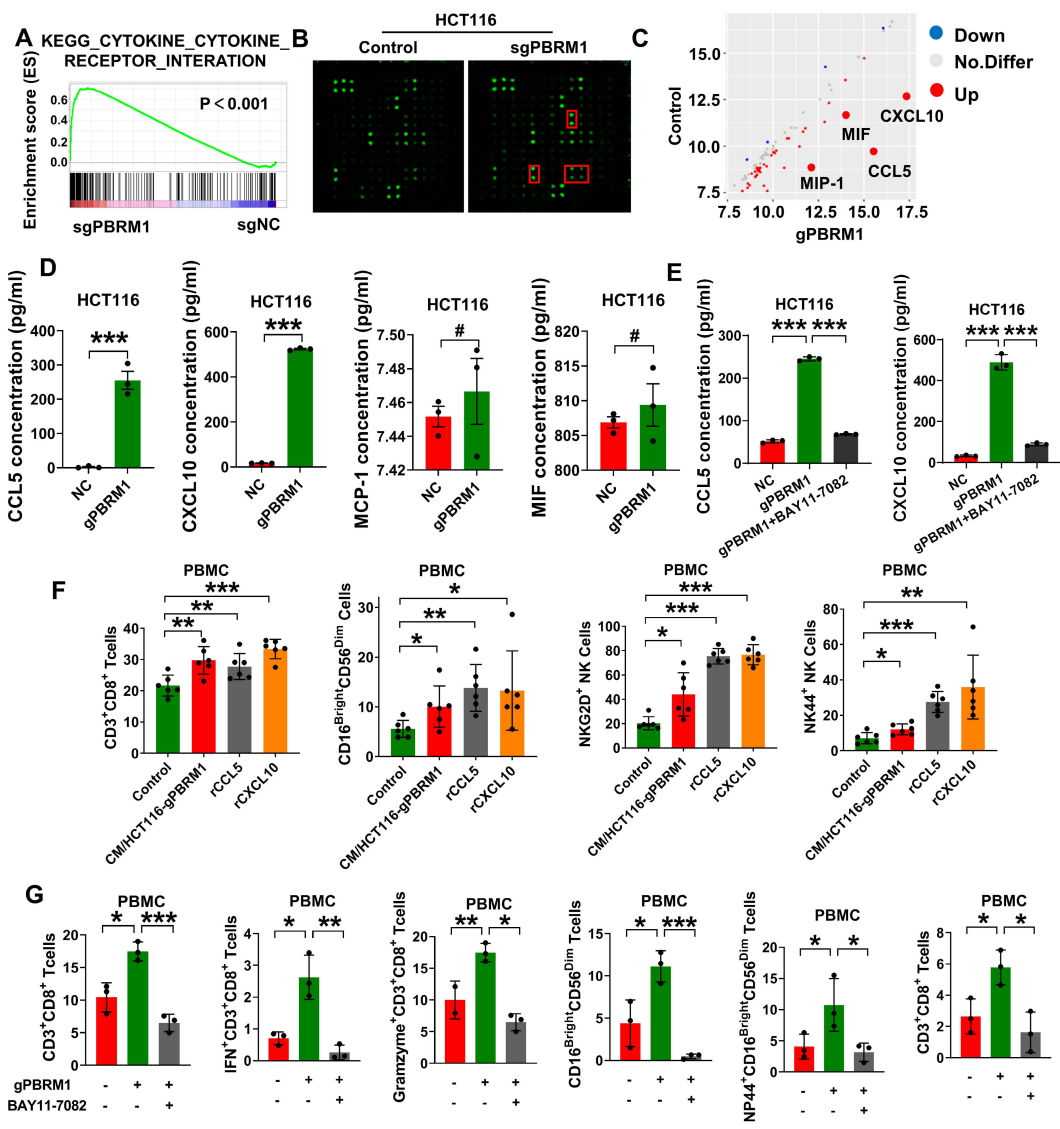
** PBRM1 mutation-induced cytokines secretion promotes CRC cell sensitivity to anti-PD1 therapy via the NF-κB signaling pathway.** (A) GSEA for cytokine _cytokine-receptor interaction pathway genes in sgPBRM1 versus sgNC HCT116 cells. n = 3 biologically independent samples per group. (B) The Human Cytokine Antibody Array was applied to detect the changes of inflammatory factors in conditioned media (CM) of sgPBRM1 and sgNC HCT116 cells. (C) The Scatterplot shows the differentially secreted factors in sgPBRM1 versus sgNC HCT116 cells. Red dots represent increased secretion by cells in the sgPBRM1 group, and blue dots represent decreased secretion in the sgPBRM1 group. Labeled are the four factors with the maximum amount of secretion and the most significant differences. (D) ELISA assay for cytokines in CM of sgPBRM1 and sgNC HCT116 cells. (E) ELISA assay for cytokines in CM of sgPBRM1 and sgNC HCT116 cells with or without BAY11-7082 (5 μM). (F) Flow cytometry of the proportion of CD8^+^ T cells and killer NK cells in PBMC after co-culture with tumor cells with or without cytokine addition. The asterisk (*) indicates P < 0.05. The double asterisk (**) indicates P < 0.01. The quadra asterisk (****) indicates P < 0.0001. (G) Flow cytometry of the proportion of CD8^+^ T cells and killer NK cells in PBMC after co-culture with tumor cells with or without BAY11-7082 (5 μM).

RNA sequencing studies indicated the possible involvement of Pbrm1 mutation in the secretion of cell growth cytokines and survival factors (Figure 5A). Hence, we used the Human Cytokine Antibody Array to detect changes in cytokine secretion in the culture medium of HT116 PBRM1 mutant and control cells (Figure 5B). As shown in Figure 5C, PBRM1 mutation significantly increased the secretion of chemokines, such as CCL5, CCL10, MIF, and MIP-1. ELISA confirmed that the secretion of CCL5 and CXCL10 was increased in the PBRM1 mutant cell line (Figure 5D) and could be inhibited by the addition of BAY11-7082, an inhibitor of the NF-κB signaling pathway (Figure 5E). The cytokine secretion could promote an increase in the proportion of CD8^+^ T cells and cytotoxic NK cells in PBMCs *in vitro* (Figure 5F), which could be reversed by applying the inhibitor to the co-culture system in PBRM1 mutant CRC cells (Figure 5G). The results suggested that the PBRM1 mutation promotes the release of cytokines, such as CCL5 and CXCL10, by activating the NF-κB signaling pathway and increasing the CD8^+^ T and cytotoxic NK cell ratio in the microenvironment, thus promoting PD1 antibody therapeutic sensitivity.

### PBRM1 deficiency promoted exosome secretion to improve CRC cell sensitivity to anti-PD1 therapy

**Figure 6:**
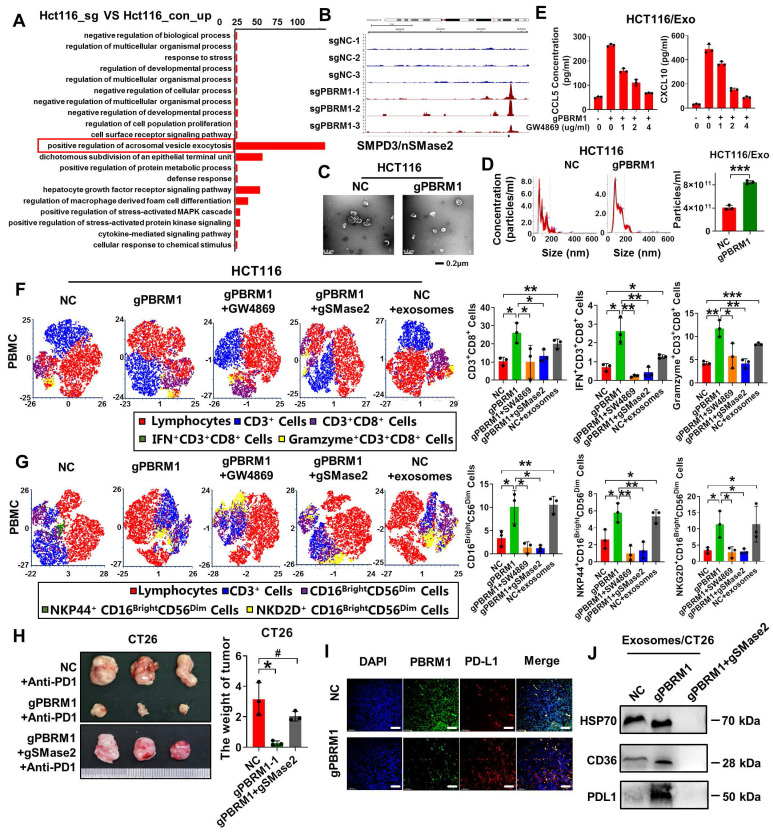
** PBRM1 deficiency promoted exosome secretion to improve CRC cell sensitivity to anti-PD1 therapy.** (A) A dot map showing the top 20 terms in Gene Ontology (GO) analysis of differential genes in sgPBRM1 versus sgNC HCT116 cells. n = 3 biologically independent samples per group. (B) IGV plots depicting ATAC-Seq signals at selected gene loci. Peak height is positively correlated to accessibility. Genome-scale is included, and the y-axis is consistent for all loci. (C) Electron microscopic demonstration of exosomes in CM of sgPBRM1 and sgNC HCT116 cells. (D) Left panel: Nanoparticle tracking analysis (NTA) of exosomes in CM of sgPBRM1 and sgNC HCT116 cells. Right panel: Histogram of NTA results. The quadra asterisk (****) indicates P < 0.0001. (E) ELISA assay for cytokines in CM of sgPBRM1 and sgNC HCT116 cells with or without BAY11-7082 (5 μM). (F) T-distributed Stochastic Neighbor Embedding (t-SNE) indicating the proportion of immune cells in PBMC after co-culture with tumor cells with or without exosomes or SW4869. (G) Bar charts show the proportion of CD8-positive T cells and NK cells in PBMC after co-culture with HCT116-NC, HCT116-sgPBRM1, and HCT116-sgPBRM1/gSMase2 with or without exosomes or SW4869. The asterisk (*) indicates P < 0.05. The double asterisk (**) indicates P < 0.01. The quadra asterisk (****) indicates P < 0.0001. (H) Left panel: sgPBRM1 and sgPBRM1/nSMase2 CT26 cells were injected subcutaneously into the backs of BALB/C mice with Anti-PD1 (200 µg/ip) to evaluate tumor growth. A representative figure of the tumors is shown. Right panel: Histogram of tumors' weight. The asterisk (*) indicates P < 0.05. The quadra asterisk (***) indicates P < 0.001. (I) Immunofluorescence assay for PDL1 in mouse tumors with sgNC and sgPBRM1 CT26 cells. Scale bar represents 50 µm. (J) Western blot analysis of PDL1 in exosomes of sgNC, sgPBRM1, and sgPBRM1/nSMase2HCT116 cells.

Studies have shown that exosomes specifically target immune cells in the microenvironment. The ATAC sequence showed that the vesicle secretion-related pathway and vesicle secretion essential motif-SMPD3 were activated in PBRM1 mutant cells (Figures 6A & B). SMPD3, which encodes SMase2, is often thought to regulate intraluminal vesicle outgrowth and exosome release in the multivesicular bodies by a mechanism not dependent on the endosomal sorting complex required for transport. Electron microscopy and nanoparticle tracking analysis showed increased secretion of exosomes in the supernatant of PBRM1 mutant cells (Figures 6C & D). After applying the exosome secretion inhibitor GW4869, the increased secretion of cytokines, such as CCL5 and CXCL10, was reversed and correlated with the inhibitor gradient (Figure 6E), suggesting that cytokine secretion depends mainly on exosomes.

Next, to investigate whether the PBRM1 mutation regulates the tumor microenvironment through exosome secretion, we constructed a colorectal tumor cell line with PBRM1 and SMase2 mutations. In the CRC and PBMCs, applying SW4869 or co-mutation with SMase2 in the PBRM1 mutant cells reversed the effect of PBRM1 mutation in promoting CD8^+^ T and cytotoxic NK cells in PMBC. More importantly, adding exosomes from PBRM1 mutant cells to control cell supernatant increased the proportion of CD8^+^ T and NK cells in PBMC. Similar results were obtained using *in situ* mouse tumor tissues. Compared with the single PBRM1-deficient group, mice with double-deficient cells treated with the PD1 antibody were similar to the control group. Concomitantly, immunohistochemistry showed increased PDL1 within the tissue microenvironment of PBRM1-deficient mice (Fig. 6I). Western blotting also confirmed elevated PDL1 in the exosomes of PBRM1-deficient CRC cells following treatment with the PD1 antibody. In contrast, this phenomenon disappeared after the SMase2 knockdown (Figure 6E).

### PBRM1 inhibitor ACBL1 promoted colorectal tumor organoid sensitivity to anti-PD1 therapy

The prevalence of PBRM1 deficiency is only about 10% in colorectal cancer. In this study, we introduced an effective and synergistic PROTAC (proteolysis-targeting chimera) degrader, PBRM1: ACBL1.

**Figure 7:**
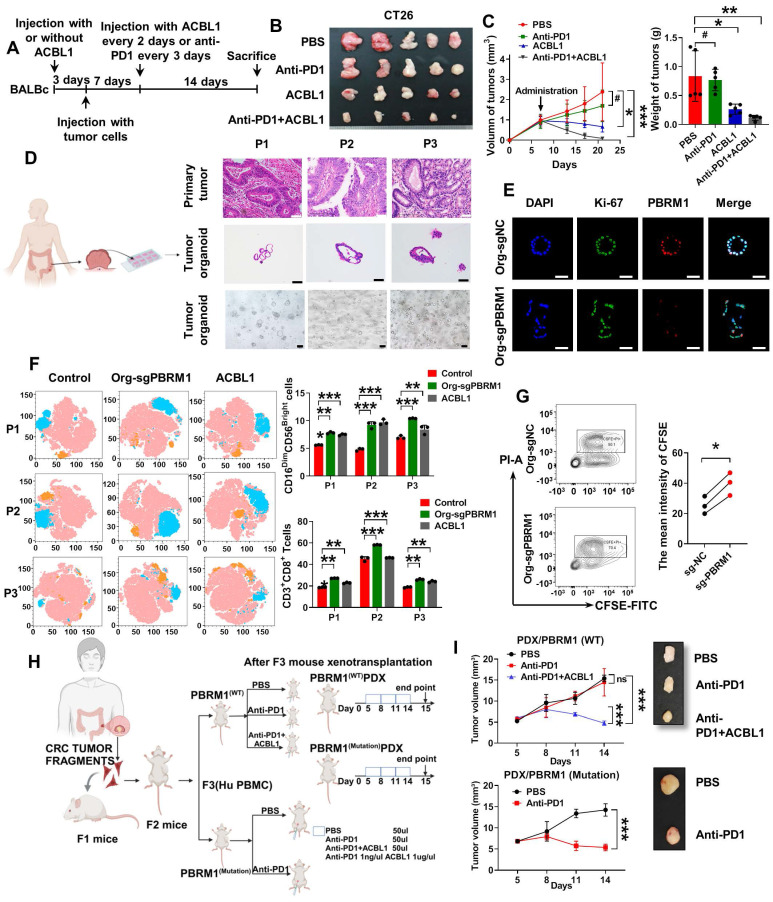
** The PBRM1 inhibitor ACBL1 promotes colorectal tumor organoids' sensitivity to anti-PD1 therapy.** (A) Flowchart of subcutaneous injection of CT26 cells and administration of Anti-PD1 (200 µg/ip) or ACBL1 (100 µg/ip) in BALB/C mice. (B) A representative figure of the tumors is shown. (C) Left panel: Growth curves of mouse tumors. Right panel: Histogram of tumors' size and weight. The asterisk (*) indicates P < 0.05. The quadra asterisk (**) indicates P < 0.01. (D) Left panel: Flowchart for constructing CRC patient-derived tumor organoids (PDTOs). Right panel: HE images of primary tumor, organoid and light microscopy images of three cases of organoid construction representatively. Scale bar represents 50 µm. (E) Immunofluorescence (IF) analysis of organoid transfected with lentiviruses encapsulating sgNC and sgPBRM1. Scale bar represents 100 µm. (F) Left panel: T-distributed Stochastic Neighbor Embedding (t-SNE) indicating the proportion of immune cells in PBMC after co-culture with PDTOs with or without ACBL1(2nmol/ul) respectively. Right panel: Bar charts showed the proportion of CD8-positive T cells and NK cells in PBMC after co-culture PDTOs with or without ACBL1(2nmol/ul). The double asterisk (**) indicates P < 0.01. The quadra asterisk (***) indicates P < 0.001. (G) 5, 6- carboxyfluorescein diacetate succinimidyl ester/propidium iodide (CFSE/PI) staining for detecting the killing ability of PBMC after co-culture with PDTOs. (H) Flowchart of two cases of patient-derived xenografts (PDXs) construction. (I) Left panel: Growth curves of PDXs. Right panel: A representative figure of the tumors is shown.

In an *in situ* tumor model constructed in BALB/C mice (Figure 7A), the application of ACBL1 in the PBRM1 wild-type group yielded results similar to those of the PBRM1 mutant group, where both promoted immune sensitization to PD1 antibodies in mice (Figure 7B and Figure 7C). We also performed long-term administration of the drug. After 42 days of PD-1 antibody treatment (200 µg/ip) or the PBRM1-ACBL1 inhibitor (100 µg/ip) given to BALB/c mice, as shown in Figure S9, the *in situ* tumor volume of the cecum of mice grown with CT26-gPBRM1-1 was not significantly changed compared with the control group; however, the tumor volume in the PBRM1 mutant group was considerably reduced after PD-1 antibody treatment. Also, ACBL1 treatment in control mice significantly enhanced the sensitivity to PD1 antibody treatment compared to the group without ACBL1 application. More importantly, after a more extended period of drug application, no apparent adverse effects were observed in the liver and kidney tissues of the mice in either the experimental or control groups.

We collected fresh tissues from three colorectal cancer cases and constructed patient-derived tumor organoids (PDTOs). As shown in Figure 7D, three PDTOs were successfully constructed and verified to have the same phenotype as the original tissues by HE and immunofluorescence staining. All tissue samples from PDTOs expressed wild-type PBRM1. Furthermore, we knocked down the expression of PBRM1 in PDTOs by virally loaded sgRNA transfection. Immunofluorescence confirmed a successful PBRM1 knockdown in PDTOs (Figure 7E). We detected the cell death index by co-culturing the edited PDTOs in autologous PBMCs and found that PBMCs had a higher lethality for PBRM1 knockdown PDTOs than the control group (Figure 7G). After 48 h of co-culture of PDTOs with autologous PBMCs, flow cytometric assays showed significantly higher numbers of CD8^+^ T cells and killer NK cells in PBMCs from the PBRM1 knockout group than in the control group (Figure 7F). The uniformity of genetic background was ensured by constructing the patient-derived xenograft (PDXs) mouse model (Figure 7H). Two cases with different PBRM1 expressions were applied to PDXs, and the humanized PBMC mice (hu-PBMC) were used in model construction to ensure the consistency of genetic backgrounds. As displayed in Figure 7I, the application of ACBL1 in the PBRM1 wild-type group yielded similar results to those of the PBRM1 mutant group, both promoting the sensitivity of mice to the PD1 antibody.

## Discussion

PD-1/PD-L1 immunotherapy has achieved a remarkable curative effect in the treatment of malignant tumors, such as renal clear cell carcinoma, melanoma, and non-small cell lung cancer. However, as the results of various clinical trials have been updated, the mechanism of action has become far more complex than that of the immune brake 16.

In this study, we screened genes associated with PD1 immunotherapeutic sensitivity by whole exome sequencing (WES) and examined the contribution of mutation frequencies in the top 12 genes, including PBRM1, SLIT1, ROBO2, and others. PBRM1, an essential component of the SWI/SNF chromatin-remodeling complex, is important in regulating chromosome accessibility. The SWI/SNF complex is present in all eukaryotic cells and regulates various processes, including cell development, differentiation, proliferation, DNA repair, and tumor suppression 17. PBRM1 is expressed at low levels in numerous tumors, including colorectal cancer, and positively correlates with the abundance of immune cells in the mesenchyme. Moreover, *in vivo* and *in vitro* experiments showed that the PBRM1 mutation did not affect the proliferation of CRC cells; however, it significantly promoted their sensitivity to PD1 antibody treatment.

PBRM1, also known as chromatin remodeling complex protein, encodes protein BAF180 involved in coding for SWI/SNF components. Previous studies have suggested that deletion of PBRM1 promotes the development of clear renal cell carcinoma 18. It has been reported that mutations in the PBRM1 gene resulted in melanoma cells being more sensitive to the T-cell-produced interferon IFN-γ and recruited more anti-tumor T-cells into the tumor periphery, resulting in increased therapeutic sensitivity to PD1 immune-surveillance point inhibitors 19. Similarly, in a cohort study, 90 patients with renal clear cell carcinoma were treated with anti-PD1 therapy 20, and tissue samples were analyzed by whole-genome chromosome sequencing. The results showed that overall survival and disease-free survival were significantly prolonged after immunotherapy in 62% of cases with loss of PRBM1 gene function.

Another study of rhabdomyosarcoma 21 reported that PBRM1 knockdown decreased immunosuppressive cytokine secretion. PBAF-PBRM1 levels in tumor samples were negatively correlated with cytotoxic T-cell infiltration and clinical prognosis, which may be associated with aberrant DNA methylation of genes regulating neuronal and kidney development. The study concluded that in tumors, such as melanoma and renal clear cell carcinoma, individuals with somatic PBRM1 mutations have increased sensitivity to treatment with PD1/PDL1 inhibitors, which might be associated with aberrant chromosomal methylation and TNF-α release. Consistent with these findings, our study found that PBRM1 deficiency in colorectal cancer enhances immunotherapy response, suggesting that PBRM1 may be an important biomarker and a potential therapeutic target for stratifying patients.

It has been reported that in ccRCC, PBRM1 promotes increased chromosome accessibility by regulating nucleosome dissociation 22, whereas in rhabdomyosarcoma, PBRM1 mutation may regulate cytotoxic T-cell infiltration by modulating aberrant chromosomal methylation 21. We examined the chromosome accessibility of tumor cells by ATAC-Sequence and found that the central peak of PBRM1-deficient CRC cells increased compared with the control group. The RNA-sequence analysis showed that the chromosome open region was enriched in proteins, such as vesicle secretion regulation, cytokine-receptor binding, the NF-κB signaling pathway, and other pathway proteins closely related to tumor development and microenvironmental immune cell regulation. These findings suggested that PBRM1 deletion can increase chromosome accessibility and chemotaxis of immune cell infiltration, thereby regulating the sensitivity of CRC cellular immunotherapy.

In recent years, it has been reported that the activation of the NF-κB signaling pathway is closely related to tumor immunity 23. Activation or dampening of the NF-κB/IRF1 axis in dendritic cells has been demonstrated to affect the recruitment and activation of anti-tumor CD8^+^ T cells by influencing the IFN-γ response and the consequent recruitment and activation of anti-tumor CD8^+^ T cells in melanoma 24. Meanwhile, Traf3 was found to regulate MHC-I through the NF-κB signaling pathway, promoting T cell-mediated tumor cell killing to promote tumor immunotherapy sensitivity 25. Using a combination of ATAC and RNA sequencing, we found that chromosome-opening sites after PBRM1 deletion were concentrated in the RELA motifs. Immunofluorescence and Western blot analyses also indicated a stimulatory role for the PBRM1 mutation in the nuclear translocation of p65 and cytoplasmic translocation of IKK-α.

Furthermore, we dissected the contribution of PBRM1 deletion in CRC by regulating inflammatory factors, such as CCL5 and CXCL10 chemotaxis, and activating cytotoxic T cells and killer NK cells, which express NKG2D in the tumor microenvironment through activation of the NF-κB signaling pathway. However, the mechanisms by which PBRM1 affects chromosomal accessibility and constructs dynamic interactions with histones, transcription factors, and active chromatin remodeling factors to maintain the stability of chromatin accessibility are not clear. In the future, mechanistic studies are needed to correlate bioinformatics analyses with functional experiments. Different working models are used to reconstruct chromosomal accessibility, such as passive competition between TF and core histones, cis-remodeling of chromatin by proximal linker histone displacement, trans-remodeling of chromatin by accessible distal regulatory elements, and direct binding of pioneer transcription factors to nucleosome DNA 26.

Besides the autocrine or paracrine mechanisms, cytokines transported and secreted by exosomes can be selectively taken up by cytokine receptor-positive cells in specific tissues, resulting in an altered immune landscape of the tissue. Higher levels of CCL2 and IL-6 were found in the exosomes of breast cancer patients, and the uptake of these cytokine-carrying exosomes by organs such as the liver and spleen was much higher than non-carrier exosomes 27. Similarly, in pancreatic ductal adenocarcinoma, exosome-borne MIF could drive tumor cells to colonize the liver by acting on Kupffer cells in the liver tissue to produce TGF-β and recruit macrophages 28.

Our study demonstrated increased exosome secretion in PBRM1 mutant CRC cells and exosomes carrying cytokines, such as CCL5 and CXCL10. More importantly, in the absence of exosome secretion, induction of T cells and NK cells by PBRM1 mutation was diminished. During PD1 antibody therapy, we also found increased PDL1 in the exosomes of cells from the PBRM1 mutant group. Moreover, PBRM1 was positively correlated with PD-L1 expression in CRC tissue samples. Although the mechanism of exosomal PD-L1 in tumor immunity is unclear, its evaluation as an indicator of anti-PD1 efficacy would be interesting. It has been suggested that exosomal PD-L1 is related to T-cell reactivation during immunotherapy and reflects successful anti-tumor immunity triggered by anti-PD-1 therapy 29.

**Figure 8:**
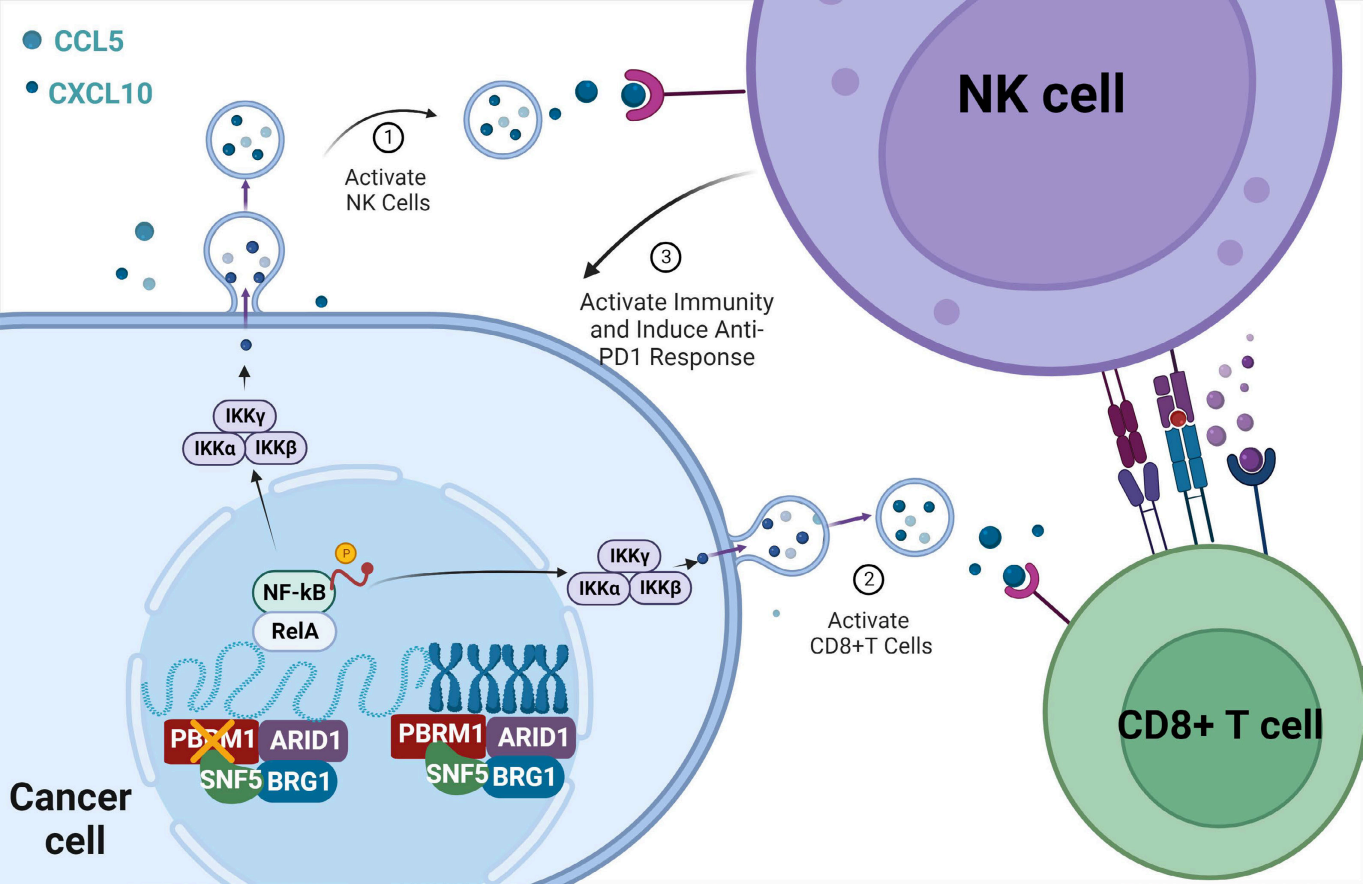
The sketch map of the regulation and mechanism of PBRM1-mediated Chromosome accessibility and CRC immunotherapy sensitivity.

In conclusion, our study examined PBRM1, a gene associated with PD1 therapeutic sensitivity in colorectal cancer, by whole-exome sequencing and found that its deficiency induced CD8^+^ T cell and NK cell infiltration in the microenvironment by regulating chromosomal accessibility, activating the NF-κB signaling pathway, and vesicle secretion (Figure 8). Our study identifies PBRM1 as a potential molecular target for enhancing immunotherapy in colorectal cancer.

## Supplementary Material

Supplementary materials and methods, figures.
