# Supplementary material for: PBRM1 deficiency enhances PD1 immunotherapeutic sensitivity via chromosomal accessibility in colorectal cancer
Source: Theranostics. 2025 Feb 18;15(8):3316–31. doi: 10.7150/thno.100793 (PMC11905143; doi:10.7150/thno.100793)
Supplement: Supplementary file 1 — Supplementary materials and methods, figures. [file thnov15p3316s1.pdf]

Supplementary Materials and Methods

Genome editing using CRISPR-Cas9

Guide sequences were designed according to previously published protocols. Guide RNAs (sgRNAs) targeting the exons of human PBRM1 or SMARD2 were designed using Benchling (https://www.benchling.com/). Double-stranded oligonucleotides containing the guide sequences were individually subcloned and cloned into the lentiCRISPR v2 plasmid according to published literature (Addgene plasmid #52961), while the PBRM1 gene was mutated by the simultaneous introduction of two independent guide sequences. Cells were transiently transfected with lentiCRISPR v2 encoding non-targeting sgRNAs or a pool of four independent PBRM1 or SMARD2-targeting sgRNAs. Twenty-four hours after transfection, the cells were selected using 1 μg ml−1 puromycin for three days. Single cells were sorted using flow cytometry and cultured. Western blotting was performed to examine the knock-out efficiency.

ATAC-seq and analysis

ATAC-seq was performed as previously described[20]. Briefly, 40,000 cancer cells were washed in cold PBS and resuspended in cytoplasmic lysis buffer (CER-I from the NE-PER kit, Invitrogen, cat. no. 78833). This single-cell suspension was incubated on ice for 5-8 min (depending on the cell line) with gentle mixing by pipetting every 2 min. The lysate was centrifuged at 1,300g for 5 min at 4 °C. Nuclei were resuspended in 50 μl of 1× TD buffer, then incubated with 2-2.5 μl Tn5 enzyme for 30 min at 37 °C(Illumina). Samples were immediately purified using a Qiagen

MinElute column and PCR-amplified with NEBNext High-Fidelity 2X PCR Master Mix (NEB; cat. no. M0541L) following the original protocol33. QPCR was used to determine the optimal PCR cycle to prevent over-amplification. The amplified library was further purified by1X NEBNext High-Fidelity PCR Master Mix (New England Biolabs, MA, USA). ATAC-seq libraries were sequenced using Illumina Novaseq 6000. Mapping of pair-end reads. Before read mapping, clean reads were obtained from the raw reads by removing adaptor sequences. Clean reads were aligned to the reference genome sequences using the BWA program. We calculated the fragment sizes for the read pairs using a BAM file from the paired-end sequencing. Several regions were sampled depending on the size of the genome and the number of processors used to estimate the summary statistics of fragment lengths. Properly paired reads were used for the computation. The bam file generated by the unique mapped reads was used as an input file, using macs2 software for call peak with a cutoff q value < 0.05.

RNA-seq and analysis

RNA was extracted from 40,000 tumor cells using the TRIzol (Thermo Fisher Scientific, 15596018). The library was constructed using the NEBNext UltraTM RNA Library Prep Kit (NEB #E7490) and the RNA-seq libraries were purified using beads (AMPure XP system). Sequencing was performed using the NovaSeq 6000 and PE150.

Quality distribution plots and base content distributions were generated using FASTQC. STAR was used to align the clean reads to the reference genome. STAR

outperforms aligners by a factor of >50 in mapping speed, while simultaneously improving alignment sensitivity and precision. In addition to the unbiased de novo detection of canonical junctions, STAR can discover non-canonical splices and chimeric (fusion) transcripts, and is also capable of mapping full-length RNA sequences.

Western blot analysis

Immunoblot analysis of cell lysates (20–60 mg) in RIPA buffer was carried out to assess protein expression in the presence of rabbit antibodies against PBRM1 (CST, 38439, 1:500), PD-L1 (Proteintech, 28076-1-AP, 1:300), phospho-NF-κB p65 (CST,3033, 1:1000), phospho-IκBα (CST, 2895, 1:1000), phospho-IKKα/β (CST, 2697, 1:1000), IκBα (CST, 4814, 1:1000), IKK-α (CST,11930, 1:1000), and β-tubulin (Abcam, 56676,1:1000).

Immunofluorescence and Immunohistochemistry (IHC)

Immunofluorescence staining was performed as previously described. Polyclonal rabbit primary antibodies against PBRM1 (CST, 38439, 1:500), PD-L1 (Proteintech, 28076-1-AP, 1:300), HRP-conjugated secondary antibody, and DAB staining kit (CWBIO, Beijing, China) were used in the experiments.

IHC was performed to investigate the protein expression in human colorectal cancer tissues. The Sections were incubated overnight with primary antibodies against PBRM1 (CST, 38439, 1:500) at 4 °C. In the present study, the sections were reviewed double-blind by three pathologists. Based on staining intensity, IHC staining was semi-quantitatively categorized as 0 (no staining), 1 (weak staining, light yellow), 2

(moderate staining, yellowish brown), and 3 (strong staining, brown). IHC scores of 2 and 3 were considered overexpression, and scores below 2 were considered low expression. Discrepancies (<5%) were resolved by simultaneous reevaluation. The significance of the correlations was determined using Pearson's $\chi 2$ test.

Supplementary Figure and Figure legends

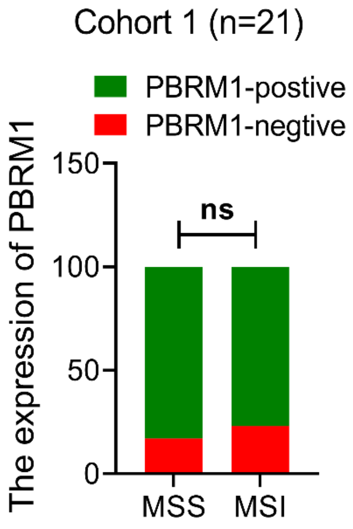

Figure S1. Statistical graph showing the relationship between PBRM1 protein expression and microsatellite status. The ns indicates P > 0.05.

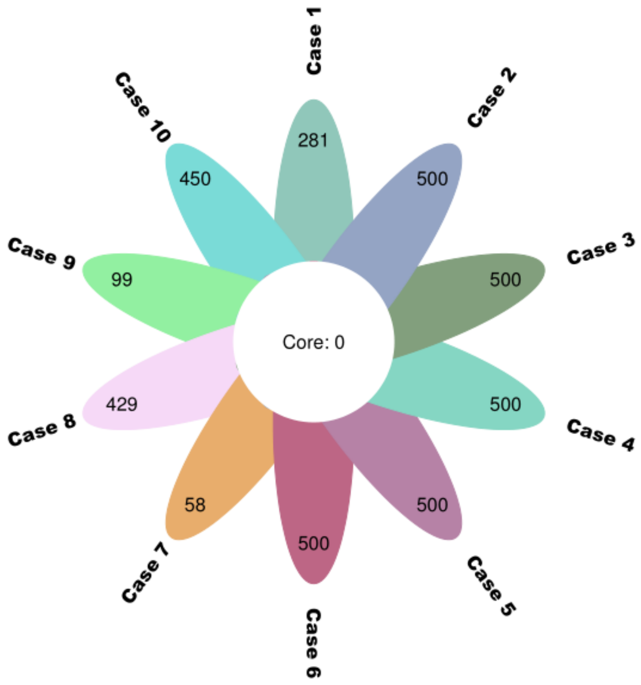

Figure S2. Flower plot showing intersection of high-frequency mutated genes in 10 CRC cases exhibiting PBRM1 mutations.

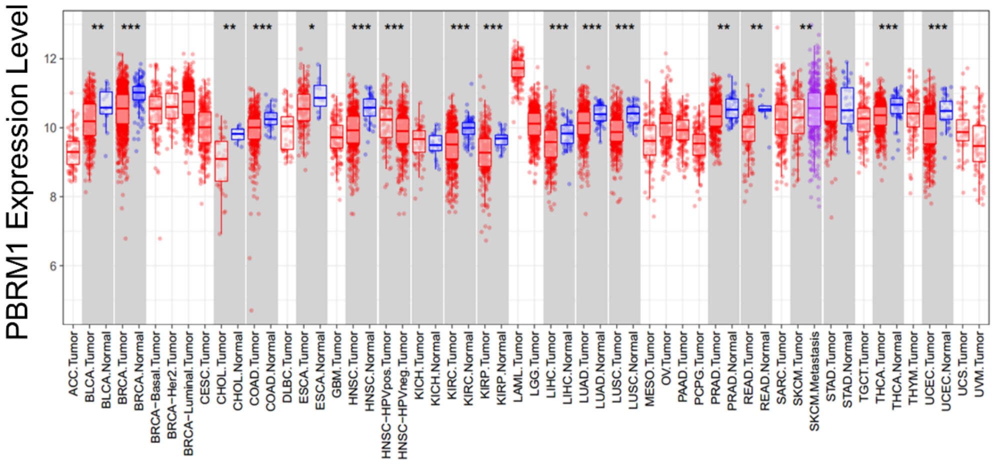

Figure S3. TIMER database analysis of PBRM1 expression in multiple tumors and paired normal tissues.

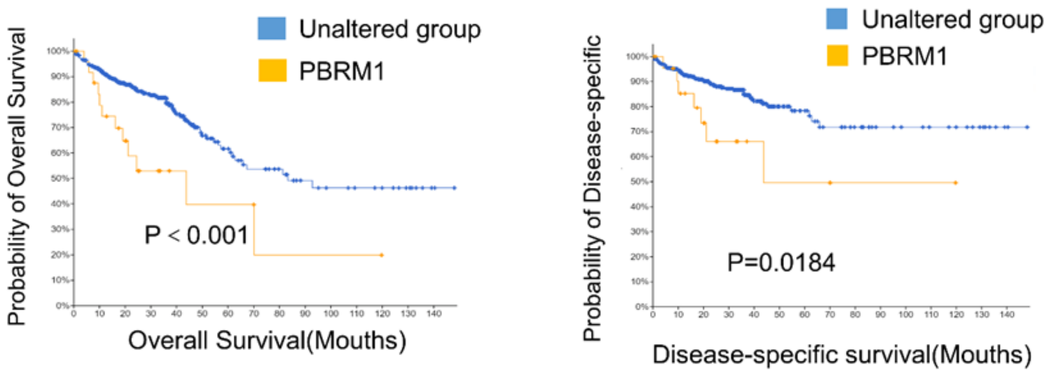

Figure S4. Kaplan–Meier survival curves and univariate analyses (log-rank) for CRC patients with or without PBRM1 deficiency.

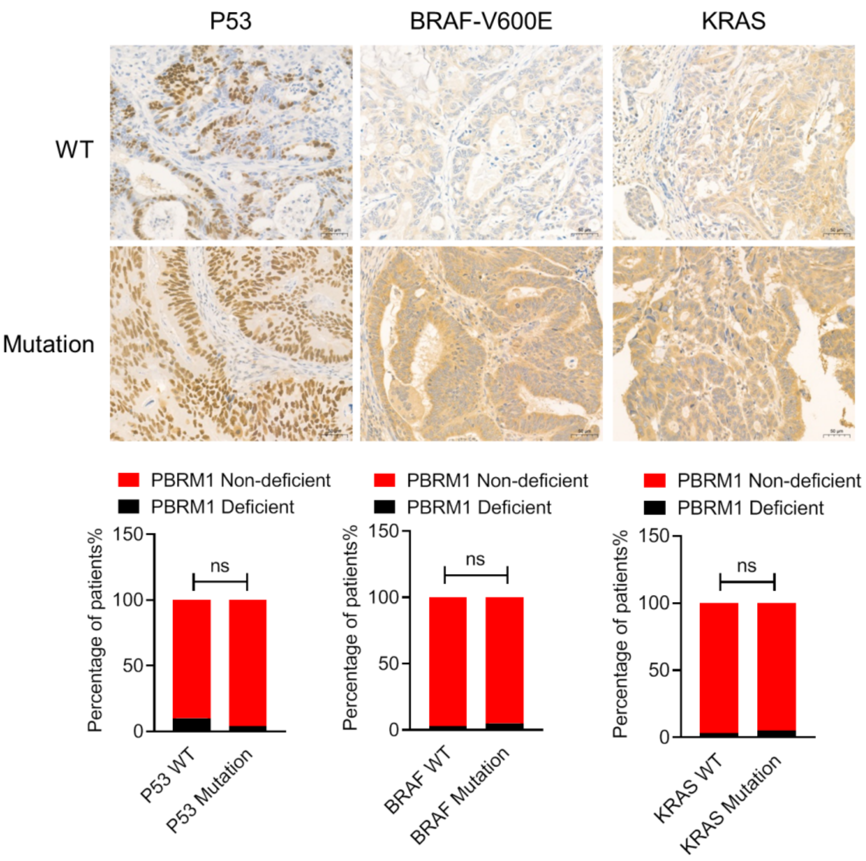

Figure S5. Immunohistochemical analysis of association between PBRM1 expression and the expression of P53, BRAF-V600E and KRAS in 61 cases of CRC tissues with paired normal tissues.

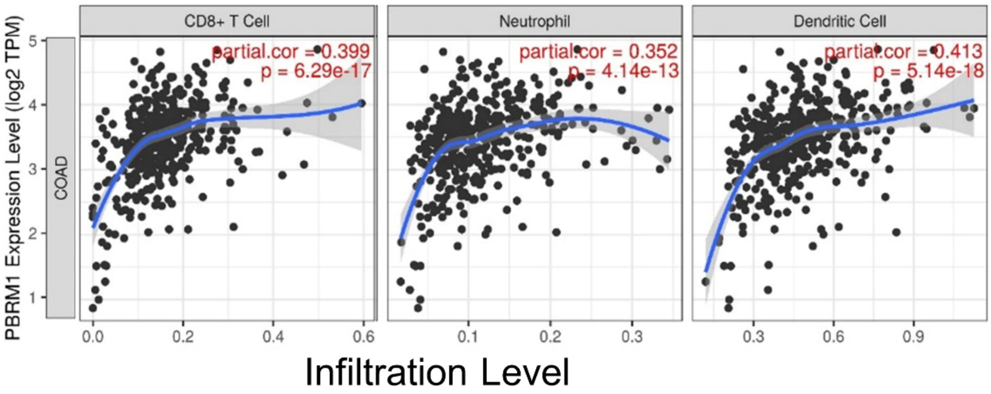

Figure S6. TIMER database analysis of the relationship between PBRM1 expression level and lymphocyte infiltration in tumor mesenchyme.

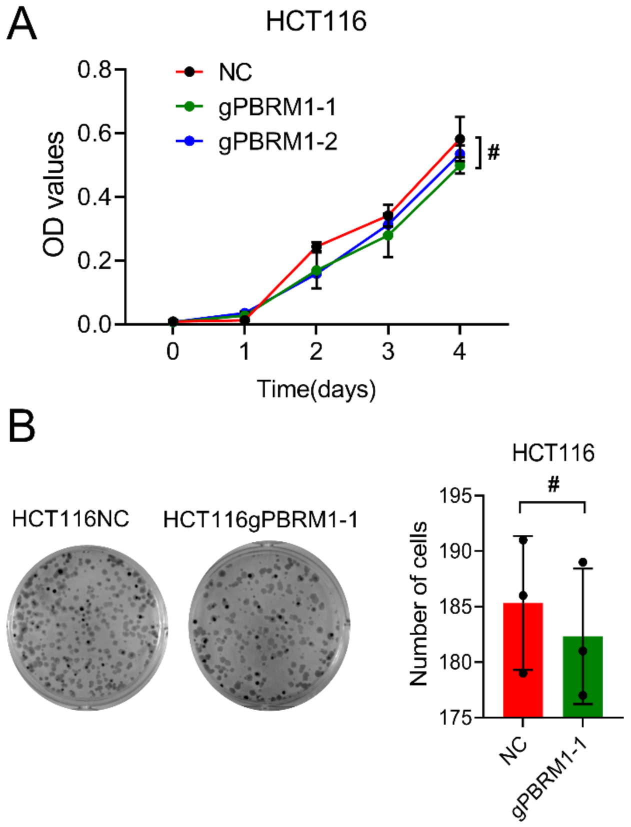

Figure S7. CCK8 analysis (A) and plate cloning assays (B) showed cell proliferation in PBRM1 mutant and control group in HCT116 cells.

HCT116-NC VS HCT116-sgPBRM1

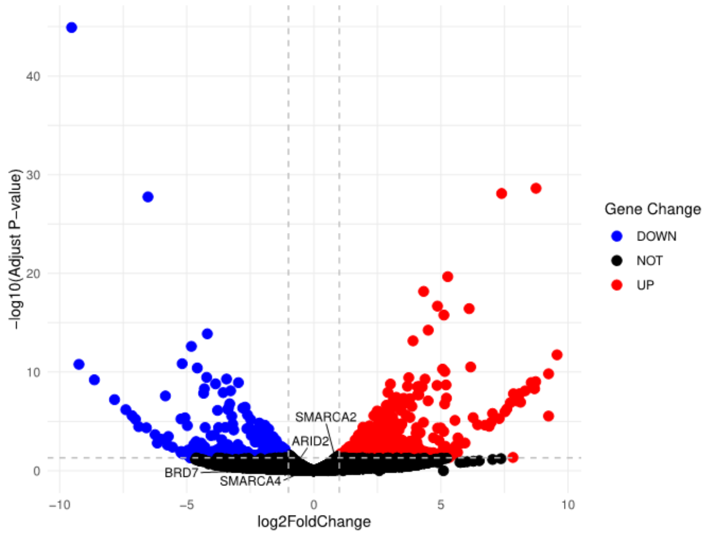

Figure S8. Volcano plot showed the transcript levels of the constitutive proteins in the PBAF complex, including BRD7, ARID2, SMARCA2, and SMARCA4 by RNA-sequence in HCT116-NC and HCT116-sgPBRM1 cells.

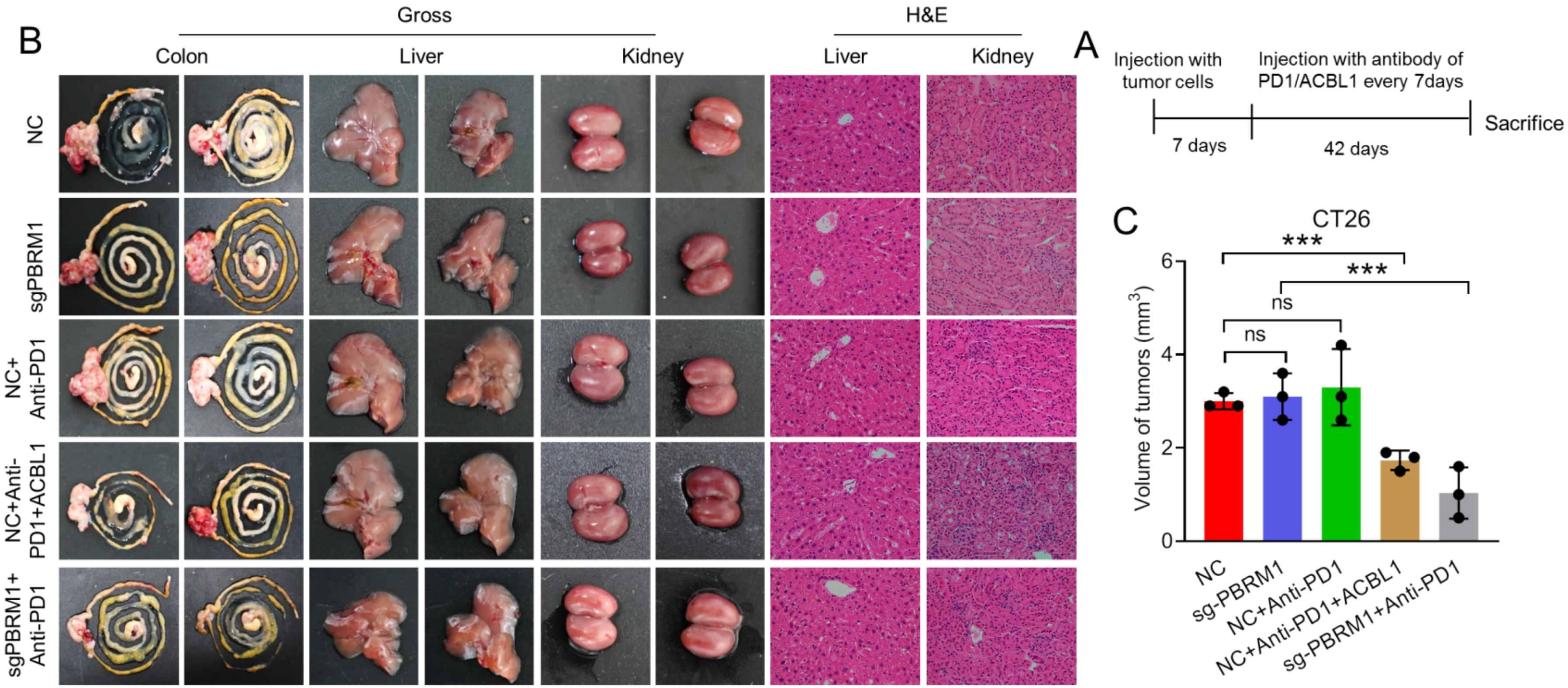

Figure S9. (A) sgNC and sgPBRM1 CT26 cells were injected into the cecum mesentery of BALB/C mice with or without Anti-PD1 (200ug/ip) and ACBL1 (100ug/ip) to assess the weight of in situ tumor and drug effects in the liver and kidney in a long-term treatment (42days). (B) A representative figure of the tumors, livers and kidneys was shown. (C) The histogram of the volume of tumors.
